# Anisotropy in Polyurethane Pre-Insulated Pipes

**DOI:** 10.3390/polym11122074

**Published:** 2019-12-12

**Authors:** Lucía Doyle, Ingo Weidlich, Marcus Illguth

**Affiliations:** Infrastructural Engineering, HafenCity University, 20457 Hamburg, Germany; lucia.doyle@hcu-hamburg.de (L.D.); marcus.illguth@hcu-hamburg.de (M.I.)

**Keywords:** cell anisotropy, polyurethane foam, sandwich structure, district heating, multiaxial loading

## Abstract

The polyurethane foam in district heating pre-insulated pipes has a critical role to play both as thermal insulation and as load bearing element, as it serves as bond between the medium pipe and the casing. Hence, knowledge on how the foam behaves under multiaxial stresses is of great importance for the design as well as for aging predictions of the network. It is known that cell shape anisotropy in polymeric foams leads to anisotropy in its mechanical properties. In this study, we evaluate and quantify the microstructural anisotropy of PU foam from pre-insulated pipes as well as its mechanical behaviour under compression in the three orthogonal directions. We cover rigid and flexible PU foam, batch and continuous manufacturing, and different pipe diameters. The results were compared with those predicted by available rectangular and Kelvin cell shape models. We have found that PU from pre-insulated pipes is orthotropic and present stronger anisotropy than that typically found in PU slabs. The traditional bonded pipes under consideration behaved in a similar way. However, when comparing the two flexible pipes in this study, despite no significant differences in cell shape anisotropy were found, a significantly different behaviour for the E modulus ratio was observed. This shows that while the manufacturing process exerts the main influence on cell shape anisotropy, to explain the difference in stiffness behaviour other factors need to be taken into consideration, such as cell size and cell size variability.

## 1. Introduction

**Figure 1:**
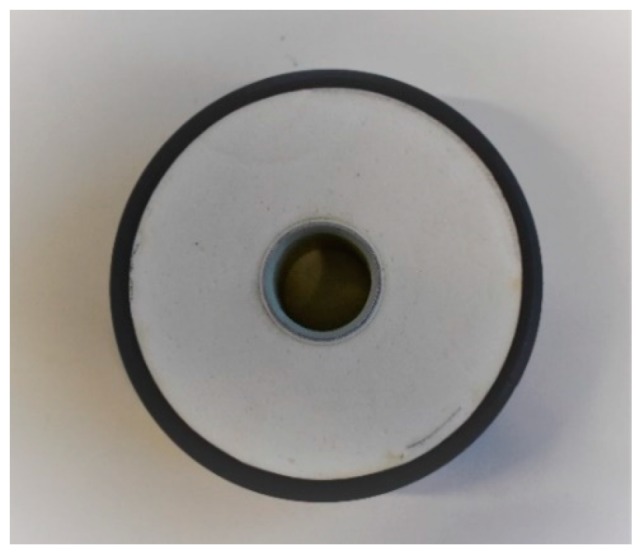
Cross-section of a PU pre-insulated pipe.

District heating (DH) pre-insulated pipes are composed of an inner medium pipe, polyurethane (PU) insulating foam, and a polyethylene (PE) casing. The PU foam bonds the medium pipe and the casing. The medium pipe can be steel or plastic (i.e., PEX) depending on the network operating temperature. Available PU pre-insulated bonded pipes can be manufactured with different processes and PU formulations. Rigid bonded steel pipes are the most widely used. They are manufactured in batch, where the PU is injected between the service pipe and the casing. Service pipe and casing are manufactured in a separate process. The flexible pipes, which have appeared in the market more recently, are manufactured in a continuous process where the PU is poured into a moving casting mould, and the PU material flows around the moving pipe. Then, the PE outer casing is extruded in place [1]. Pre-insulated flexible pipes are available, presenting different degrees of flexibility achieved through different formulations of the PU foam and with corrugated and smooth casing. Figure 1 presents a cross-section of a pre-insulated pipe.

DH pipe networks are usually buried underground. They are subjected to multiaxial loading, as the operation temperature produces an expansion of the pipe, which is partially restrained by the surrounding soil. This expansion leads to axial shear stress on the foam, as the axial expansion is partially counterbalanced by the soil friction, and in compression of the foam in the radial direction due to the earth pressure. Hence, the polyurethane foam in this application has a critical role to play both as thermal insulation and as load bearing element, minimizing the heat losses of the network, critical for the sustainability of the whole district heating system, while serving as bond between the medium pipe and the casing. Therefore, knowledge on how the foam behaves under multiaxial stresses is of great importance both for the design as for aging predictions of the network, which is key as this infrastructure’s lifecycle is expected to last for over 30 years. However, details on the microstructure and behaviour of the PU foam in DH pre-insulated pipes are insufficiently well known, and therefore design standards and calculation methods used in the sector relate mainly to the steel medium pipe [2,3]. While aging of PU foam in district heating pipes has been a subject of research during recent years, the focus has been placed mainly on oxidation and thermal degradation [4,5,6], but details on the microstructure and its relation to the bonded pipe’s mechanical behaviour remain mostly unexplored. In order to optimize structural design, detailed understanding of the mechanical behaviour of the elementary materials is required. There is a need for better characterization of the PU foam used in DH pipes, which would allow the development of material models and numerical simulations that could support the design process.

The mechanical properties of cellular solids are greatly influenced by the microstructure of the foam, as well as the properties of the solid material constituting it. Key structural features are [7]:

- The relative density.
- The degree to which cells are open or closed.
- The geometric anisotropy of the foams.

It is a noted phenomenon that the cells of polymeric foams tend to be elongated in the foam rise direction of the mould due to the acting viscous forces during the foaming process, and are hence anisotropic. This phenomenon and its impact on the material’s properties is well described in [7].

The PU foam used in DH pipe insulation presents closed-cells. The first model relating the shape-anisotropy to the mechanical properties of cellular foams is proposed by Huber and Gibson [8] as an extension of the model proposed by Gibson and Ashby [9]. This model is based on a simple cubic cell geometry. The cell aspect ratio *R*, would influence the elastic modulus of the cell foam according to:

(1) $$\frac{E_{3}}{E_{2}}=\frac{E_{3}}{E_{1}}=\frac{2R^{2}}{1+{\left(\frac{1}{R}\right)}^{3}},$$

where *E*_3_ is the elastic modulus in the rising direction, *E*_1_ and *E*_2_ in the perpendicular direction to the foam rise, and *R* is the anisotropy ratio, defined as the ratio of the largest cell dimension to the smallest.

This expression assumes axisymmetric cells. For the case of orthotropic cells, where all three dimensions of the cell differ, the different values of *R* are needed to characterize it:

(2) $$\frac{E_{1}}{E_{2}}={\left(R_{12}\right)}^{2}\left[\frac{1+{\left(R_{32}\right)}^{3}}{1+{\left(R_{31}\right)}^{3}}\right],$$

(3) $$\frac{E_{2}}{E_{3}}={\left(R_{23}\right)}^{2}\left[\frac{1+{\left(R_{13}\right)}^{3}}{1+{\left(R_{12}\right)}^{3}}\right],$$

(4) $$\frac{E_{3}}{E_{1}}={\left(R_{31}\right)}^{2}\left[\frac{1+{\left(R_{21}\right)}^{3}}{1+{\left(R_{23}\right)}^{3}}\right]$$

For closed cell foams, an additional term should be considered [7]:

(5) $$\left(1-\phi \right)\frac{2R}{1+\left(\frac{1}{R}\right)},$$

where Φ is the fraction of solid material located in the cell’s struts, which for open cell foams is φ = 1. However, closed-cell foams tend to mechanically behave similar to open-cell ones when the membranes across the cell faces are thin in relation to the cell edges [8]. Since measurement of cell wall thickness is difficult [10], we will assume the PU under study behaves mechanically, such as an open cell foam.

Later models relating the mechanical properties to the cell anisotropy have been developed for open cell foams using an elongated tetrakaidecahedron Kelvin model [11] as the repeating unit cell, such as those from Gong et. al. [12], Ridha et. al. [13] and Sullivan et al. [14]. This geometry more closely represents the cellular structure observed in polymer foams than the rectangular cell. The fundamental difference between the Kelvin model developed by Sullivan et al. from those by previous authors is that the geometry and size of the repeating unit cell is defined by three independent dimensions, allowing to account for additional variation in the unit cell shape. The equivalent expression to Equation (1) with this model would be:

(6) $$\frac{E_{3}}{E_{1}}=\frac{E_{3}}{E_{2}}=\frac{R}{4}[\frac{\left(2\tilde{Q}R^{2}+\frac{64Q^{3}}{\sqrt{16+{\tilde{Q}}^{2}R^{2}}}\right)C_{1}+\frac{8R{\tilde{Q}}^{3}C_{2}\left(32+4Q\sqrt{16+{\tilde{Q}}^{2}R^{2}}\right)}{\left(4Q+2\sqrt{16+{\tilde{Q}}^{2}R^{2}}\right)\left(16+{\tilde{Q}}^{2}R^{2}\right)}\left(\frac{\rho_{f}}{\rho_{s}}\right)}{1+\left(\sqrt{3}-\pi /2\right)+\frac{8R^{3}{\tilde{Q}}^{5}\left(\frac{20\sqrt{3}-11\pi }{2\sqrt{3}-\pi }\right)}{\left(4Q+2\sqrt{16+{\tilde{Q}}^{2}R^{2}}\right)\left(16+{\tilde{Q}}^{2}R^{2}\right)}\left(\frac{\rho_{f}}{\rho_{s}}\right)}]$$

where

$$\tilde{Q}=2+\sqrt{2}Q,$$

*ρ_f_* = density of the foam
*ρ_s_*= density of the basis solid material

for an hypocycloid:

C1=3−π2,

$$C_{2}=\frac{20\sqrt{3}-11\pi }{2\sqrt{3}-\pi },$$

Q=b/(L cos θ),

where *b*, *L* and *θ* are dimensions describing the cell shape. For more insights on the geometric description of the elongated tetrakaidecahedron unit cell, the reader is referred to [14]. It should be noted that this unit cell is axisymmetric.

The anisotropy of polyurethane foams and its impact on their mechanical properties has been extensively studied [8,15,16,17,18,19,20]. However, significant differences and variability of the foam and the obtained results are expected between these studies and the case of PU pre-insulated pipes, due to:

(a) The foam manufacturing process can have a great influence on the resulting microstructure of the foam [21]. All previous studies have been conducted with PU slabs foamed in rectangular moulds, where the distance between the mould walls is significantly larger than in pre-insulated pipes. Since cell anisotropy in foams is caused by the acting viscous forces between the liquid and the mould walls during the foaming process [7], it is expected that the narrower distance between mould walls in the case of the pre-insulated pipes will have a higher impact on the anisotropy of the cells. Moreover, the geometry of the mould, annular in the case of pipes, could have an influence on the cell’s microstructure. Furthermore, the effects of manufacturing in a continuous process remains to be explored.
(b) PU foams can be tailored through modifications in the chemical formulation [22,23,24]. However, details on the chemical formulation of the PU are rarely documented in the studies found in the literature and they may or may not match those of PU insulated pipes.
(c) Not all studies cover the three orthogonal directions.

This paper seeks to address some of the challenges faced in the network design, damage accumulation and aging modeling for district heating piping systems by closing the knowledge gap on the microstructure and mechanical anisotropic behavior of the PU insulating foam from batch-produced bonded pipes and continuously produced flexible pipes.

## 2. Materials and Methods

In this study, three different types of pipes where investigated: traditional bonded pipes with steel medium pipe, rigid PU foam and smooth PE casing, flexible bonded pipes with PEX medium pipe, flexible PU foam and PE corrugated casing (denoted FC-DN40 in this study), and flexible bonded pipe with PEX medium pipe, flexible PU and PE smooth casing (denoted FS-DN40). For the traditional bonded pipe, three nominal diameters were evaluated: DN20 (denoted B-DN20), DN40 (B-DN40) and DN100 (B-DN100). The flexible pipes’ nominal diameter was DN40. All pipes were insulation series 1 (insulation thickness 28.5 mm) manufactured by Logstor.

The traditional bonded pipes used in this study were manufactured in a batch process by injecting the PU insulating foam between the service pipe and the outer casing. The casing is manufactured in a previous process and one pipe is manufactured at a time [25]. The PU foam is blown with cyclopentane, with properties as required by EN 253 [26]. Further information about the PU formulation is not provided by the manufacturer.

The flexible pipes are manufactured in a continuous process, where the PU is poured in a moving casting mould, hence the PU material flows around the moving pipe. Then, the PE outer casing is melted in place in an extruder station [1], manufactured according to EN 15632-1 [27] and EN 15632-2 [28]. The formulation of the PU from the two flexible pipe types included in this study is visibly different, however further information about the chemical formulation is not provided, as it is proprietary data from the manufacturer. Flexible pipes are supplied in coils of up to 200 m length, which makes the laying of the pipes faster and more economical. Flexible pipes with smooth casing are typically used for branches. The flexible pipes with corrugated casing have a small bend radius as to allow for laying of the pipe on difficult sites and around obstacles. This extra flexibility is achieved through the geometric design of the corrugated casing and the chemical formulation of the PU [25].

### 2.1. Sample Preparation

**Figure 2:**
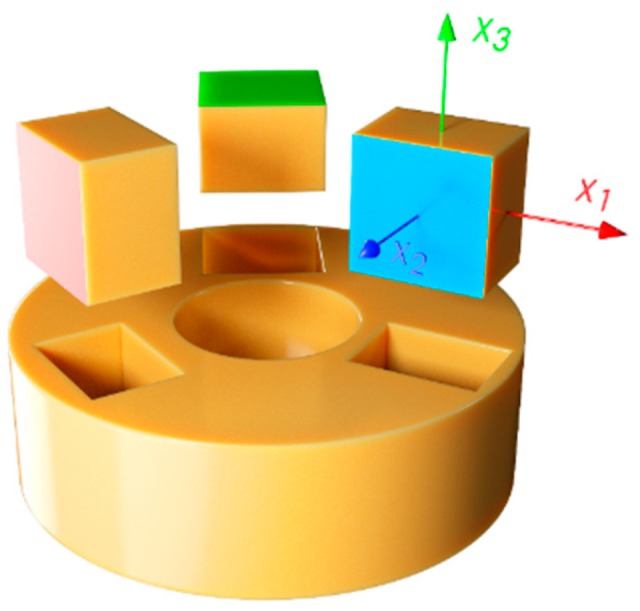
Sample extraction procedure.

Samples were machined out of the pipes following [26] as far as possible. The pipes were stored at 23 °C for at least 72 h prior removal of the casing. After discarding 500 mm of the pipe ends, cuboids were cut out of the pipe’s insulation according to Figure 2 with different orientations, in order to mechanically test the foam along the three orthogonal directions X_1_ (red), X_2_ (blue) and X_3_ (green). Sample size was 30 × 30 × 20 mm for the B-DN100 and ca. 25 × 25 × 20 mm for the other pipes, since the smaller diameter prevented the extraction of larger specimens. However, successful testing of samples this size under uniaxial compression can be found in studies in the literature [19,29], and given the obtained 1000 times cell size to sample size difference, it can be assumed that the used sample size will have no impact on the results [30]. While it was foreseen in the design of experiments to extract three samples of each case equally distributed around the circumference, this was not possible as the tolerances in pipe dimensions from the manufacturing process made the pipes slightly oval, preventing the extraction samples of the same size from all segments of the circumference.

### 2.2. Microstructural Characterization

The cross sections of all five pipe foams were examined in an optical microscope (Leica DMLP, Wetzlar, Germany). Slices of the PU foam where cut along the three orthogonal directions under study for each pipe with a cutter (planes 1-2, green; 1-3, blue and 2-3, red, see Figure 2). As to facilitate the view of the cells through the microscope, two sample preparation procedures were followed and compared: the first was infiltrating the samples with a blue coloured epoxy resin under vacuum. After curing the resin, the samples were polished until reaching the cell walls. The second procedure consisted in shading the surface of the foam with a black felt tip pen. While both procedures resulted valid, the simplicity of the second favoured this technique.

The cell size and shape were then measured from the obtained micrographs by adjusting the cells to an ellipse using Fiji [31]. The shape anisotropy *R* can then be calculated as the ratio from the largest dimension to the shortest. The rotation angle of the ellipse was measured in order to confirm a preferential direction of the cell elongation. Around 100 cells were measured per cross section and pipe.

### 2.3. Mechanical Characterization

Standard [32] was followed as far as possible. The main deviation is in the smaller sample size used, as described and justified in Section 2.1. Five specimens per pipe type and orientation were tested for compression using a universal testing machine, under a displacement controlled rate of 2 mm/s. The force was measured with a 20 kN load cell, accuracy class 0.5 (HBM, Darmstadt, Germany). The strain was measured by 3D digital image correlation (DIC) [29,33] using an ARAMIS 5M adjustable stereo camera system (GOM mbh, Braunschweig, Germany) with a resolution of 2448 × 2051 pixels. The images were acquired at frequency of 1 Hz. The strain measured by the ARAMIS optical system’s software is based on a stochastic pattern recognition analysis. Therefore, a stochastical pattern was painted on one side.

Samples where individually accurately measured using caliper and weighed prior testing.

Engineering stress-strain curves where derived from the obtained data. The E modulus is obtained for each case from the slope of the initial linear segment of the curves. Given that the E modulus is the property most sensitive to cell shape [7], ratios of *E*_3_/*E*_1_ and *E*_3_/*E*_2_ are related to the shape anisotropy ratio *R* for each pipe type and compared with the available models. For completeness of the study, the compressive stress at 10% strain (σ_10_) was obtained, as its value is a requirement included in EN 253 [26].

## 3. Results

### 3.1. Microstructure of the PU Foam

**Figure 3:**
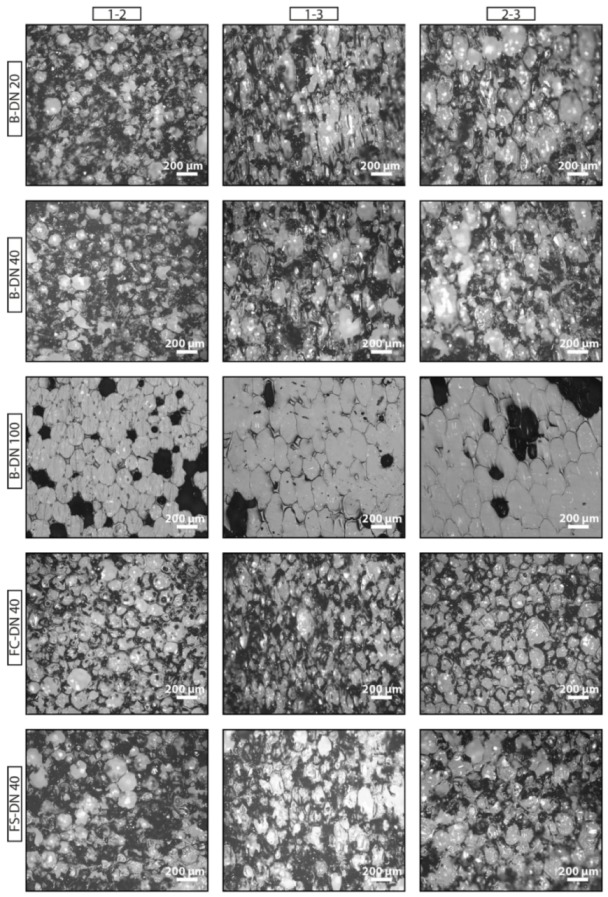
Foam micrographs. Pipe types are displayed in the different rows and the three orthogonal planes in columns.

Micrographs of the three sections of the PU foam for the five pipes under consideration are presented in Figure 3. The obtained average cell size and shape anisotropy ratio *R* is presented in Table 1, as well as the obtained cell symmetry.

**Figure 4:**
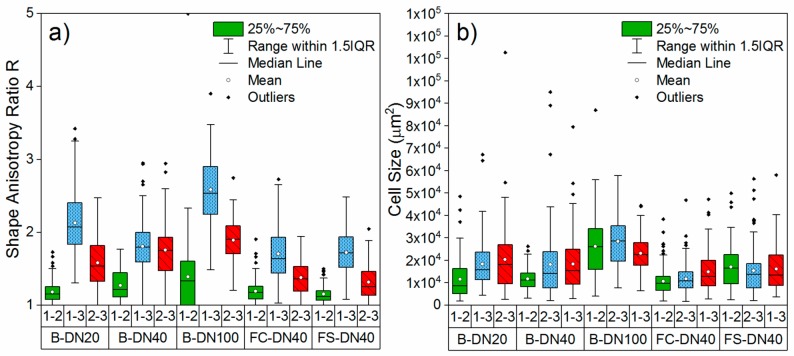
Distribution of measured *R* (**a**) and cell size (**b**) per plane and pipe type.

The obtained *R* distribution and cell size for each plane is plotted in Figure 4 for each case.

### 3.2. Mechanical Behaviour of the PU Foam

**Figure 5:**
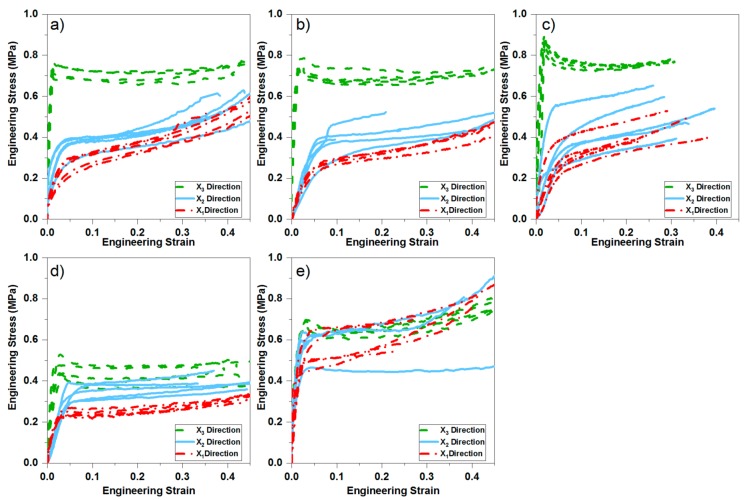
Stress–Strain curves for (**a**) B-DN20 (**b**) B-DN40 (**c**) D-DN100 (**d**) FS-DN40, and (**e**) FC-DN40.

The obtained E modulus and σ_10_ per pipe type and compression direction is presented in Table 2. The resulting engineering stress-strain curves for the PU foams tested under uniaxial compression in the three orthogonal directions are presented in Figure 5a–e. The shape of the obtained curves corresponds well with those expected for polymeric foams: an initial linear elastic region, which is controlled by cell wall bending of the cells, followed by a plateau associated with the collapse of the cells [7].

Significant differences can be found between the samples deformed in the three orientations. Curves from samples deformed in the X_3_ direction (rising of the foam) present a pronounced peak in the stress at the outset of plastic instability, followed by a stress drop and a flat plateau region. The shape curves from samples deformed in the X_1_ and X_2_ directions do not present this pronounced peak. The shape of the curves from pipes B-DN20, B-DN40, B-DN100 and FS-DN40 agree with the typical load-deflection curves for rigid polyurethane foam, and the FC-DN40 for elastomeric polyurethane [7]. Hence the foam from FS-DN40 could be classified as semi-rigid. It can be seen from Figure 5a–c that the three traditionally bonded pipes behave in a similar way, with a much higher compressive strength in the X_3_ direction, followed by X_2_ and X_1_, while FS-DN40 (Figure 5d) presents a less pronounced difference between the compressive strength for the different directions, although following the same tendency. Comparing the two flexible pipes through Figure 5d,e, a different behaviour is observed. The stress strain curves in the three directions overlap for FC-DN40, despite having no significantly different shape anisotropy ratio than FS-DN40.

**Figure 6:**
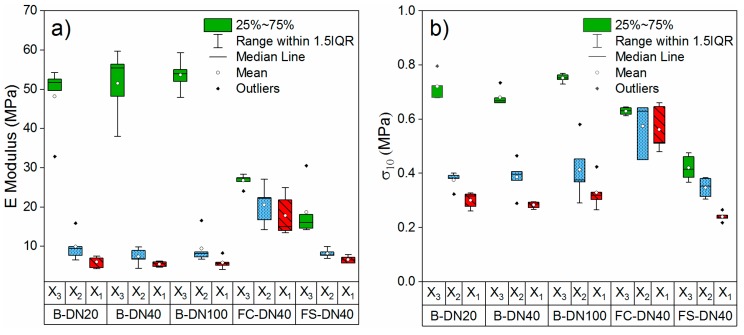
Distribution of the obtained E modulus (**a**) and σ_10_ (**b**) per direction and pipe type.

The Young’s modulus E where obtained from the initial slope of the stress-strain curve for each tested specimen and are presented in Figure 6a. The compressive strength at 10% strain is presented in Figure 6b.

All pipes present mechanical anisotropy in the E modulus under compression and the compressive strength between the X_3_ direction and X_1_ and X_2_.

## 4. Discussion

### 4.1. Microstructure of the PU Foam

Elongation of the cells in the rise direction is easily observed from Figure 3 and Figure 4. For the plane 1-2, despite diverging from circularity (which would correspond with an aspect ratio of 1), the percentage of cells elongated in the same direction is in the range 56% to 74%, while in the 2-3 and 1-3 planes they are in the range 97% to 100% in the case of the bonded pipes. Therefore, it is clear that the aspect ratio of plane 1-2 shows irregularity of the cells’ shape, while in the planes 2-3 and 1-2 shows strong anisotropy in the foam rise direction, which is axial to the pipe length (X_3_). As for the flexible pipes, in which due to the continuous manufacturing process the foam expands both around the pipe diameter as well as axial to the pipe length as the pipe is pulled through the extruder, we find anisotropy of the cells in the axial direction (X_3_), showing that this is the predominant expansion direction. However, statistical evaluation of the cell geometries shows that the extent of the anisotropy is lower for both flexible pipes than for the traditional bonded pipes. Given that both flexible pipes have different PU formulations, densities and casing geometry but present no significant difference in R, we can conclude that the manufacturing process exerts the main influence on the observed shape anisotropy ratio.

Literature reports a typical *R* for polymeric foams of around 1.3 [7]. The study on PU by [8] yielded an *R* between 1.2 and 1.6, while that of [20] ranged from 1.34 to 1.72. In our study, we have obtained an *R* from 1.31 for the case of FS-DN40 up to 2.58 for the B-DN100 bonded pipe. This shows that the PU foam in pre-insulated pipes presents a much stronger anisotropy than PU slabs. This can be explained by the geometry of the mould, the annular section of the pipe in this case. When foaming from a liquid in a mould, viscous forces act between the mould walls and the foaming melt as the volume expansion leads it to rise in one direction, provoking an elongation of the cells [7]. The distance between walls in the bonded pipe is of roughly 28 mm, while the foam expands along the 6 m length of the pipe. Therefore, the acting viscous forces are higher than those in a rectangular mould where the distance between walls is larger in relation to the rise direction. This shows the importance of evaluating foams manufactured under representative conditions and how sandwich materials may have different properties and behaviour than their individual constituents.

In order to determine if the foams present axisymmetric or orthotropic behaviour, a series of *t*-tests with 0.05 significance level were run comparing *R* in the 1-3 and 2-3 planes for each pipe type. The results are shown in Table 1. With the exception of B-DN40, all pipes were found to present orthotropic shape anisotropy in the microstructure. Shape anisotropy ratio was found higher in the 1-3 plane, corresponding to the X_2_ direction. In this direction, the distance between the mould walls, which would be the outer diameter of the medium pipe and the inner diameter of the PE casing is smaller than in the X_1_ direction, which would be casing to casing.

#### 4.1.1. Effect of Pipe Diameter

The effect of increasing pipe diameter on the microstructural anisotropy of the cells was evaluated between the three traditionally bonded pipes under consideration. The hypothesis was that anisotropy would increase with the pipe diameter for the same insulation thickness, as the surface of the pipes increases with the diameter, and with it, the friction forces between the liquid and the mould during the foaming process, contributing to the shape anisotropy. The three pipe diameters where compared one to one in the 1-3 and 2-3 planes, using a *t*-test with 0.05 significance level. *R* was found to be significantly higher in B-DN100 compared to B-DN40 and B-DN20 in both directions, while B-DN40 was higher than B-DN20 in direction 2-3, but not in 1-3. One possible explanation is that the pipe surface difference between the DN20 and DN40 pipes would not be significant enough as to produce an increase in *R*. However, we cannot conclude that there is an evident correlation between the pipe diameter and the cell shape anisotropy for the diameter range under study.

#### 4.1.2. Comparison between Smooth and Corrugated Flexible Pipes

After running the *t*-test, no significant difference was found between the FSDN40 and FCDN40 pipes in any of the three orthogonal directions. Therefore, we can conclude that the manufacturing process has a larger impact on cell shape anisotropy than the foam formulation.

### 4.2. Mechanical Behaviour of the PU Foam and Relation to the Cell Shape Anisotropy

The PU foam of the five pipe types under study present mechanical anisotropy on the E modulus and σ_10_ under compression. The three traditional bonded pipes under study present similar E modulus and E modulus ratios. The three traditional bonded pipes and flexible pipe FS-DN40 present similar E for the X_1_ and X_2_ direction, which is the X_3,_ which varies and increases with the shape anisotropy.

It is interesting to compare the two flexible pipes. While they present no significant difference in cell shape anisotropy, their mechanical behaviour under compression is very different. The modulus ratio for FC-DN40 is roughly 50% lower than that of FS-DN40. While the difference in E modulus for the same directions can be explained by the different chemical formulation, the difference in ratio does not relate to the shape anisotropy ratio in the same way than the other PU foams under study, showing that other effects come into play. One possible explanation is the effect of cell size and cell size variability. It has been noted that the Young modulus decrease with increasing cell size variations [10]. From Figure 4b and Table 1 we can see that the variability in cell size for FC-DN40 is ~35% lower than that of FS-DN40 in planes 1-2 and 2-3, which could explain the lower E modulus for FS-DN40. It could be however argued that, since the cell sizes have been determined from 2D micrographs and cells have different sizes at different altitudes, the measured sizes might diverge from the real values. This effect would however appear equivalently in all measured planes. While the most rigorous indicator for cell size is cell volume, as applied by [34], its measurement implies 3D reconstruction and complex image processing to obtain the polyhedral profile of each cell [10]. 2D micrographs are commonly used for the determination of cell size [8,10,19,20] and the obtained cell size has been reported to be close to that measured with 3D reconstruction [35]. Another variable that can affect the E modulus is the variability in cell wall thickness [10,36], which is out of the scope of our study.

**Figure 7:**
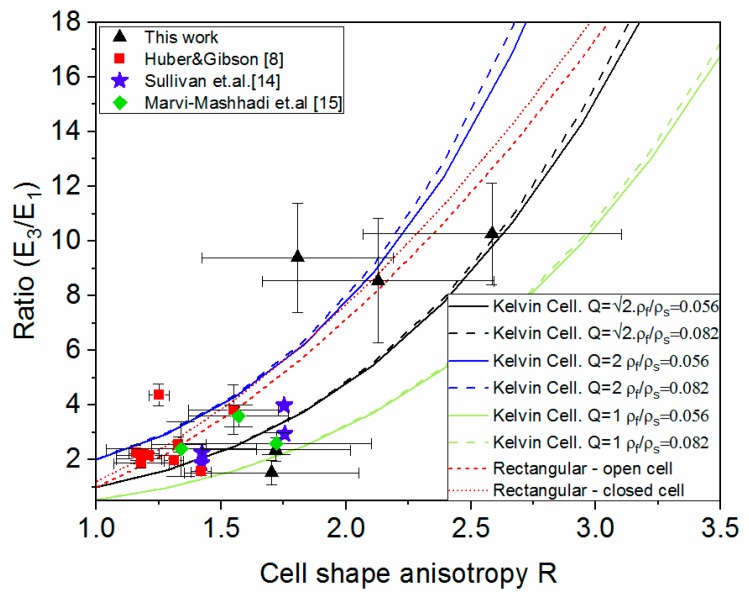
Modulus ratio vs. shape anisotropy.

In Figure 7 the modulus ratio vs. shape anisotropy *R* is plotted for the five pipe types under study, as well as values from the literature. The rectangular cell model [1] for both open-cell and closed-cell (assuming Φ = 0.8) as well as the Kelvin cell model [2] under the scenarios Q = 2, Q = $\sqrt{2}$ and Q = 1 and ρ_f_/ρ_s_ = 0.056 and ρ_f_/ρ_s_ = 0.082 (corresponding to the foam densities 67.9 kg/m^3^ and 99.2 kg/m^3^, see Table 2, and ρ_s_ = 1200 kg/m^3^ [3]). Since the literature data corresponds to axisymmetric foams and the Kelvin cell model assumes axisymmetric, only the ratios between X_3_/X_1_ are plotted to facilitate the comparison. From this graph we can observe, firstly that the shape anisotropy and the modulus anisotropy present in the traditional bonded pipes is significantly higher than the cases previously reported in the literature. This shows how foams in sandwich structures present different morphologies and behaviour than stand-alone foams and the need to characterize them individually obtained under real manufacturing conditions. As for the relation between the modulus ratio and *R*, the obtained results could be best fitted to the Kelvin cell model using different values of Q. Foam cells with similar shape anisotropy but different modulus ratio could be explained by differences in the cell shape. Still, cation needs to be taken given the experimental variability in *R* and modulus ratio. The fact that the cells in PU from DH pipes are orthotropic, and that this involves a deviation from the idealized Kelvin cell, needs to be highlighted. While unit-cell based models can yield important results, real foams are typically non-uniform presenting variations in size and shape in the struts and in the cells, limiting their accuracy.

## 5. Conclusions

Polyurethane foam in pre-insulated bonded pipes for district heating applications present strong cell shape, elastic modulus and compressive strength anisotropy, higher than that reported for PU foam slabs especially for the traditional bonded pipes. This is due to the geometry of the mould, the annular section between the medium pipe and the pipe casing, where the distance between the walls is much smaller than that of rectangular moulds for the production of slabs. This highlights the importance of the foam manufacturing in the resulting cell microstructure and how foams in sandwich structures present different properties and behaviour than foam slabs. The cells have been found to be mainly orthotropic, with different dimensions in the three orthogonal directions.

The three traditional bonded pipes under consideration behaved in a similar way. However, when comparing the two flexible pipes under consideration, no significant difference in cell shape anisotropy was found, but significantly different behaviour as for E modulus ratio. The equivalent shape anisotropy is due to the same manufacturing process. To explain the difference in stiffness behaviour other factors need to be taken into consideration, such as cell size and cell size variability.

## Figures and Tables

**Table 1:** Mean cell area and *R* per plane and pipe type and resulting symmetry.

Pipe	Plane	Number of Measured Cells	Mean Cell Area (mm^2^)	Cell Area Std. dev (mm^2^)	Mean *R*	*R* Standard Deviation	Symmetry
B-DN20	1-2	103	0.012	0.009	1.18	0.15	orthotropic
	1-3	108	0.018	0.010	2.13	0.46	
	2-3	100	0.020	0.015	1.58	0.35	
B-DN40	1-2	114	0.012	0.005	1.27	0.19	axisymmetric
	1-3	103	0.018	0.013	1.81	0.38	
	2-3	101	0.018	0.015	1.75	0.38	
B-DN100	1-2	79	0.026	0.013	1.39	0.54	orthotropic
	1-3	81	0.023	0.008	2.58	0.52	
	2-3	74	0.028	0.011	1.89	0.31	
FC-DN40	1-2	130	0.011	0.006	1.19	0.15	orthotropic
	1-3	107	0.015	0.008	1.70	0.35	
	2-3	137	0.012	0.007	1.37	0.23	
FS-DN40	1-2	100	0.017	0.010	1.15	0.12	orthotropic
	1-3	100	0.016	0.010	1.72	0.30	
	2-3	101	0.015	0.010	1.32	0.24	

**Table 2:** Obtained density, E modulus, and σ_10_ per pipe type and compression direction.

Pipe	(kg/m^3^)	Direction	E (MPa)	E Std dev (MPa)	σ10 (MPa)	σ10 St. dev (MPa)
B-DN20	76.2	X3	52.1	1.9	0.72	0.05
		X2	8.4	1.6	0.38	0.03
		X1	6.1	1.5	0.30	0.03
B-DN40	76.2	X3	51.5	8.6	0.68	0.03
		X2	7.4	2.1	0.39	0.06
		X1	5.5	0.7	0.28	0.01
B-DN100	75.6	X3	53.6	4.2	0.75	0.02
		X2	7.7	0.8	0.41	0.11
		X1	5.2	0.8	0.33	0.06
FS-DN40	67.9	X3	15.7	1.8	0.63	0.01
		X2	8.2	1.1	0.57	0.11
		X1	6.6	1.0	0.56	0.08
FC-DN40	99.2	X3	27.4	0.8	0.42	0.05
		X2	20.5	5.1	0.35	0.04
		X1	17.9	5.2	0.24	0.02
